# Tunneling nanotube (TNT)-mediated neuron-to neuron transfer of pathological Tau protein assemblies

**DOI:** 10.1186/s40478-016-0386-4

**Published:** 2016-11-04

**Authors:** Meryem Tardivel, Séverine Bégard, Luc Bousset, Simon Dujardin, Audrey Coens, Ronald Melki, Luc Buée, Morvane Colin

**Affiliations:** 1 Université Lille, Inserm, CHU-Lille, UMR-S1172, Alzheimer & Tauopathies, 59000 Lille, France; 2 Paris-Saclay Institute of Neuroscience, Centre National de la Recherche Scientifique, Université Paris-Saclay, 91190 Gif-sur-Yvette, France; 3 Massachusetts General Hospital, Neurology Department, Mass General Institute for Neurodegenerative Disease, Charlestown, MA 02129 USA; 4 Inserm UMR- S1172, JPArc, ‘Alzheimer & Tauopathies’, Place de Verdun, 59045 Lille Cedex, France

## Abstract

A given cell makes exchanges with its neighbors through a variety of means ranging from diffusible factors to vesicles. Cells use also tunneling nanotubes (TNTs), filamentous-actin-containing membranous structures that bridge and connect cells. First described in immune cells, TNTs facilitate HIV-1 transfer and are found in various cell types, including neurons. We show that the microtubule-associated protein Tau, a key player in Alzheimer’s disease, is a bona fide constituent of TNTs. This is important because Tau appears beside filamentous actin and myosin 10 as a specific marker of these fine protrusions of membranes and cytosol that are difficult to visualize. Furthermore, we observed that exogenous Tau species increase the number of TNTs established between primary neurons, thereby facilitating the intercellular transfer of Tau fibrils. In conclusion, Tau may contribute to the formation and function of the highly dynamic TNTs that may be involved in the prion-like propagation of Tau assemblies.

**Electronic supplementary material:**

The online version of this article (doi:10.1186/s40478-016-0386-4) contains supplementary material, which is available to authorized users.

## Introduction

Understanding the transmission of an infectious agent from one cell to another was a challenge of the last century. The involvement of cell-surface receptors has been shown, but other routes have also been described. Tunneling nanotubes (TNTs) form one such path. TNTs have been described in various cell types, including neuronal and immune cells. They are filamentous-actin-containing membranous structures with a diameter of 50 to 800 nm, not always linked to the substrate, and forming bridges that connect remote cells [1–6]. For instance, TNTs physically connect T cells, presenting a new pathway for HIV-1 transmission [7]. In such cells, the tip of the TNT is an active zone of actin cytoskeleton reorganization and contains ezrin, Exo70, myosin 10 and N-WASP, suggesting a regulation at the cellular level [8, 9]. Extrinsic factors such as arachidonic acid in endothelial cells [10], HIV-1 infection in macrophages [11], oxidative stress [12] and prion-like proteins (e.g., Huntingtin fibrils, TDP-43) in neuronal cells [6, 13, 14] have been shown to trigger TNT formation.

Many protein aggregates have prion-like properties: they can act as self-propagating templates. They disrupt cellular proteostasis, eventually leading to neurodegenerative disorders such as Alzheimer’s disease (AD), Parkinson’s disease (PD), amyotrophic lateral sclerosis (ALS), or transmissible spongiform encephalopathies (TSEs) [15–17]. The exact mechanisms of the cell-to-cell spreading of pathological species are still subject to intense investigation. Among others, the role of TNTs in such propagation has been suggested in Huntington’s disease, Parkinson’s disease and ALS/fronto-temporal dementia [18]. Regarding Alzheimer’s disease, the amyloid Aβ peptide has been shown to traffic through TNTs and to induce cytotoxicity [12]. The role of TNTs in aggregated Tau spreading has not yet been documented.

In the present work, using two different cellular models (CAD neuronal cells and rat primary embryonic cortical neurons), we demonstrate that extracellular Tau species acts as an extrinsic factor leading to increased formation of TNTs, which in turn facilitate the intercellular spread of pathological Tau.

## Materials and methods

**Ethics statement-** Animals were provided by Janvier Laboratories and had access to food and water ad libitum. Animal experiments were performed in compliance with and with the approval of the local ethics committee (agreement CEEA 062010R), standards for the care and use of laboratory animals, and the French and European Community guidelines.

### Cell culture

***Primary Embryonic Neuronal Culture-*** Rat primary embryonic cortical neurons (primary neurons) were prepared from 17–18-day-old Wistar rat embryos as follows. The brain and meninges were removed. The cortex was dissected out and mechanically dissociated in culture medium by trituration with a polished Pasteur pipette. Once dissociated and after blue trypan counting, cells were plated in Ibidi μ-Dishes (Biovalley) or Lab-Tek four-well chamber slides (Becton Dickinson) coated with poly-D-lysine (0.5 mg/mL) and laminin (10 μg/ml). For dissociation, plating, and maintenance, we used Neurobasal medium supplemented with 2 % B27 and containing 200 mM glutamine and 1 % antibiotic-antimycotic agent (Invitrogen). Primary neurons at 7 days in vitro (DIV7) were infected with lentiviral vectors (LVs) encoding GFP/mCherry actin, tubulin or human wild type Tau (hTau1N4R containing a V5 tag; V5-hTau1N4R).

***Cell lines-*** Mouse neuronal CAD cells (mouse catecholaminergic neuronal cell line, Cath.a-differentiated) were cultured in Opti-MEM (Invitrogen) with 10 % fetal bovine serum, penicillin/streptomycin (1 %) and L-glutamine (1 %). Neuronal CAD cells were plated overnight in poly-D-lysine (0.5 mg/mL) coated Ibidi μ-Dishes for live imaging or Lab-Tek four-well chamber slides for immunostaining. Neuronal CAD cells were infected with LVs encoding GFP-actin, mCherry-tubulin or human wild-type Tau (hTau1N4R containing a V5 tag; V5-hTau1N4R).

**Viral vectors-** The procedures to produce the lentiviral vectors (LVs) and to control their viral titers and the absence of competent retroviruses have been described previously [19]. All viral batches were produced in appropriate areas in compliance with institutional protocols for genetically modified organisms according to the “Comité Scientifique du Haut Conseil des Biotechnologies” (Identification Number 1285).

**Antibodies-** As part of this work, various primary antibodies were used: mouse anti-α acetylated Tubulin (Sigma; 1:200 for immunocytochemistry); rabbit polyclonal antibody to V5 (Merck Millipore; 1:10,000 for immunocytochemistry); rabbit polyclonal antibody against the C-terminal part of Tau (C-ter, raised in-house; 1:800 for immunocytochemistry and 1:10,000 for biochemistry) [20]; rabbit polyclonal antibody M19G, which recognizes the N-terminal part of Tau (N-ter, raised in-house; 1:10,000 for biochemistry) [21]; and rabbit polyclonal raised against human anti-myosin 10, which detects myosin 10 from multiple species, including mouse and rat (Sigma; 1:200 for immunocytochemistry). These antibodies were visualized using appropriated secondary antibodies coupled to Alexa 488 or 647 (Life Technologies; 1:1000 for Alexa 488 and 1:500 for Alexa 647).

**Immunofluorescence-** Neuronal CAD cells and primary neurons were washed with pre-warmed PBS, fixed with 4 % paraformaldehyde (PFA) for 20 min at room temperature, permeabilized with 0.2 % Triton X-100 for 20 min at room temperature and blocked for 45 min at room temperature using blocking solution (Bovine Serum Albumin (BSA) 2 % in PBS). Cells were then incubated overnight at 4 °C with primary antibody diluted in blocking solution before being carefully washed and incubated for 30 min at room temperature with the appropriate Alexa Fluor-conjugated secondary antibody. Cells were washed and mounted with VectaShield/4′,6-diamidino-2-phenylindole (DAPI, Vector Laboratories) to label nuclei.

**Tubulin tracker staining for live imaging-** Neuronal CAD cells were plated overnight at 100,000 per 35 mm glass-bottomed culture μ-dish (Biovalley, France), washed with pre-warmed PBS and incubated 30 min at 37 °C with tubulin Tracker green (Life Technology; 1:1000 dilution) diluted in HBSS buffer and rinsed 3 times with pre-warmed PBS before imaging.

**Fluorescence imaging-** Immunofluorescence and short time-lapse acquisitions were performed using an inverted confocal microscope (LSM 710, Zeiss, Jena, Germany) with a 40× oil-immersion lens (NA 1.3 with an optical resolution of 176 nm) with an optical resolution of X nm. DAPI, Alexa 488/GFP, mCherry and Alexa 647 were imaged using UV, Argon 488 nm, DPSS 561 nm and Helium/Neon lasers 633 nm. Images were processed with ZEN software. Long time-lapse acquisitions were performed using an inverted Yokogawa Spinning Disk confocal microscope with a 63× oil-immersion lens (NA 1.4 with with an optical resolution of 164 nm) and epifluorescence microscope (Eclipse Ti-E, Nikon, Tokyo, Japan) with a 40× air-immersion lens (NA 0.9 with an optical resolution of 338 nm). Images were processed with ZEN software and NIS software. To reduce noise, the signal was subjected to line averaging to integrate the signal collected over four lines. The confocal pinhole was adjusted to facilitate a minimum field depth. A focal plane was collected for each specimen. Cells were maintained at 37 °C and 5 % CO_2_ during real time acquisitions. All setups, using similar illumination and recording conditions (detector frequency, gain, and laser intensity), were applied to non-treated primary neurons to avoid misinterpretation due to non-specific labeling (Additional file 1: Figure S1 d-e).

### Human Tau1N4R purification

Full-length human Tau1N4R cDNA was cloned in the pET14b vector. hTau1N4R was expressed in *E. coli* BL21 DE3 CodonPlus cells (Stratagene). Cells were grown in LB medium to an optical density at 600 nm of 0.8 absorbance units. hTau1N4R expression was induced with 0.5 mM IPTG for 3 h. The cells were then harvested by centrifugation (4000 g, 10 min). The bacterial pellets were resuspended in lysis buffer (20 mM MES pH 6.8, 500 mM NaCl, 1 mM EGTA, 0.2 mM MgCl_2_, 5 mM dithiothreitol, 1 mM PMSF + 1 tablet of Complete (Roche)) per liter and lysed by sonication. Cell extracts were clarified by centrifugation at 14,000 g, 30 min. The lysate was heated to 80 °C for 20 min and centrifuged at 14,000 g for 30 min. The supernatant was dialyzed against 100 volumes of buffer A (20 mM MES pH 6.8, 50 mM NaCl, 1 mM EDTA, 1 mM MgCl_2_, 2 mM DTT, 0.1 mM PMSF) at 4 °C. The dialyzed protein mixture was loaded on an SP Sepharose column (60 ml bed volume). Proteins were separated with a linear gradient of 0 to 100 % buffer B (20 mM MES pH 6.8, 1 M NaCl, 1 mM EGTA, 1 mM MgCl_2_, 2 mM DTT, 0.1 mM PMSF). Fractions were analyzed via SDS-PAGE stained with Coomassie blue. Fractions containing hTau were pooled and dialyzed against 100 volumes of PBS buffer containing 1 mM DTT. The hTau concentration was determined spectrophotometrically using an extinction coefficient at 280 nm of 7450 M^−1^.cm^−1^. Pure hTau1N4R at a concentration of 50 to 100 μM. Fibrillar samples were sonicated for 5 min on ice in 2-ml Eppendorf tubes in a VialTweeter powered by an ultrasonic processor UIS250v (250 W, 2 4 kHz; Hielscher Ultrasonic, Teltow, Germany) set at 75 % amplitude, 0.5 s pulses. Aliquots were stored at −80 °C. Length distribution of sonicated Tau fibrils has been obtained by measuring the length of 227 fibrils in negatively stained TEM samples (15 to 85 nm with a mode size of 55 nm) (Additional file 2: Figure S2).

### Human Tau1N4R assembly and labeling

Fibrillation of hTau1N4R was achieved at 40 μM in the presence of 10 μM heparin by shaking 0.5 ml solution aliquots at 37 °C in an Eppendorf Thermomixer set at 600 rpm for 4 days. Fibrils were spun for 20 min at 20 °C and 16,000 rpm. The amount of fibrillar material was estimated by subtraction of the soluble fraction remaining after centrifugation from the initial concentration. The pelleted material was resuspended in PBS at an equivalent monomeric hTau1N4R concentration of 100 μM.

Labeling of hTau1N4R fibrils was achieved by the addition of 2 molar equivalents of lysine-reactive ATTO 488, ATTO 568 or ATTO 647 (Life Technologies #A20003) for 1 h at room temperature. The unreacted fluorophore was removed by two cycles of centrifugation at 15,000 g for 10 min and resuspension of the pellet in PBS.

### Sup35NM purification and assembly

Purification and assembly of Sup35NM were performed as described in Krzewska et al. [22]. Labeling of Sup35NM fibrils was performed identically to that of Tau fibrils.

### Electron microscopy

The nature of hTau1N4R and Sup35NM assemblies was assessed using a JEOL 1400 transmission electron microscope following adsorption onto carbon-coated 200-mesh grids and negative staining with 1 % uranyl acetate. The images were recorded with a Gatan Orius CCD camera (Gatan).

**TNTs activation-** For TNT activation experiments, neuronal CAD cells and primary neurons were incubated for 5 min at 37 °C with 1 μM recombinant hTau1N4R fibrils labeled with ATTO 647 or unlabeled. Rinses with pre-warmed PBS were performed (3×) before immunostaining or real-time imaging.

**Antibody saturation-** Before incubation with cells, C-ter antibodies were incubated (24 h/4 °C/orbital agitation) with blocking solution containing either saturating concentrations of recombinant hTau1N4R fibrils (100×) or BSA (100×) as a control. Cells were immunolabeled as described above.

**Uptake and transfer of hTau1N4R fibrils-** Neuronal CAD cells were plated overnight at 60,000 cells per well in poly-D-lysine-coated Lab-Tek four-well chamber slides (Becton Dickinson). Cells were infected with LVs encoding mCherry-Actin. ATTO 488-hTau1N4R fibrils were diluted at 1 μM in 100 μL of OptiMEM (Gibco). Then, 96 μL of OptiMEM and 4 μL of Lipofectamine-2000 (Invitrogen) were added to the ATTO 488-hTau1N4R fibrils to a final volume of 200 μL for 20 min before the mixture was added to the cells. For primary neurons, 50,000 cells were plated in poly-D-lysine- and laminin-coated Lab-Tek four-well chamber slides (Becton Dickinson). Cells were infected at DIV7 with LVs encoding mCherry-Actin. ATTO 488-hTau1N4R fibrils were diluted at 1 μM in 100 μL of Neurobasal medium (Gibco). Then, 96 μL of Neurobasal medium and 4 μL of Lipofectamine-2000 (Invitrogen) were added to the ATTO 488-hTau1N4R fibrils to a final volume of 200 μL for 20 min before the mixture was added to the cells. Six hours later, cells were rinsed with pre-warmed medium (3×) before immunostaining.

For neuron-to-neuron transfer, CAD cells or primary neurons (100,000 cells/dish) were plated on Ibidi μ-Dishes and infected with LVs encoding GFP-Actin. ATTO 568-hTau1N4R fibrils were diluted at 1 μM and added to the cells. Six hours later, cells were wash with pre-warmed PBS (3×) before acquisition using real time microscopy.

**Electrophoresis and immunoblotting-** Neuronal CAD cells were rinsed once in PBS and lysed in RIPA buffer (150 mM NaCl, 1 % NP40, 0.5 % sodium deoxycholate, 0.1 % SDS, and 50 mM Tris HCl; pH = 8.0). The positive controls were wild-type mouse hippocampal cell homogenate (CTL1) or neuronal CAD cells infected with LVs encoding hTau1N4R (CTL2). Protein concentrations were determined (PIERCE BCA Protein Assay Kit), and samples were diluted at 1 μg/μL in LDS containing 50 mM DTT. Then, 15 μg of protein was denatured at 100 °C for 10 min, loaded on 4-12 % NuPAGE Novex gels (Invitrogen), and transferred to nitrocellulose membranes. Membranes were blocked in Tris-buffered saline, pH 8.0, 0.05 % Tween 20 with 5 % skim milk or 5 % BSA and incubated with the appropriate primary overnight at 4 °C. Membranes were then rinsed and further incubated with horseradish peroxidase-labeled secondary antibody (goat anti-rabbit IgGs, Sigma), and bands were visualized by chemiluminescence (ECL, Amersham Biosciences) with a LAS3000 imaging system (Fujifilm).

**Statistical analysis-** Data are presented as the means (± SEM) of experiments performed at least in triplicate and are representative of the results obtained from three independent experiments that produced similar results. Statistical analyses were performed using the Mann–Whitney *U*-Test (GraphPad prism software) to determine the *p*-value. The differences were considered significant at * *p* < 0.05, ** *p* < 0.01, or *** *p* < 0.001.

## Results

### Tau is present within TNT in CAD cells under basal conditions

To facilitate reporting on our results, pseudo-colors have been used in some figures to visualize actin in red and tubulin in green (live cells). We first characterized TNTs in neuronal CAD cells following published criteria (Additional file 3: Figure S3 and Additional file 4: Figure S4): 1) the presence of actin in TNTs without tubulin [1, 2], 2) the size of TNTs and the kinetics of their formation [1, 2, 23–26] and 3) the myosin 10 labeling [8]. Extensions between neuronal CAD cells were characterized using multiple methodologies and have all properties of TNTs. They were tubulin negative and actin positive in all systems used: endogenous acetyl-tubulin immunoreactivity (Additional file 3: Figure S3 a, polymerized tubulin), tubulin tracker (Additional file 3: Figure S3 b, polymerized tubulin), overexpressed mCherry-tubulin (Additional file 3: Figure S3 c, monomeric and polymerized tubulin) and tagged actin (GFP or mCherry) (Additional file 3: Figure S3). Currently, there is no specific marker of TNTs. However, the actin-interacting protein myosin 10 has been shown to be necessary for the formation and the functionality of TNTs [8]. Confocal microscopy allowed the identification of actin-positive and tubulin-negative TNTs containing myosin 10 protein (Additional file 3: Figure S3 d) in neuronal CAD cells. These structures are easily detectable via live imaging in overexpressing systems of actin and tubulin, with a diameter of approximately 400 nm and lengths ranging from 10 to 30 μm (Additional file 4: Figure S4 a), and they are rapidly formed thanks to a “filopodia-like” (Additional file 4: Figure S4 b and d) or a “kiss-and-run” mechanism (Additional file 4: Figure S4 c and e), as previously described [24, 26, 27]. Altogether, these data showed that we have engineered all necessary tools for identifying TNTs. In contrast, the optical resolution of confocal microscopy did not allow us to image TNTs in primary neurons (*n* = 5 independent experiments; data not shown).


Movie for “filopodia-like” formation mechanism in neuronal CAD cells. (MOV 3265 kb)



Movie for “kiss-and-run” formation mechanism in neuronal CAD cells. (MOV 506 kb)


**Fig. 1:**
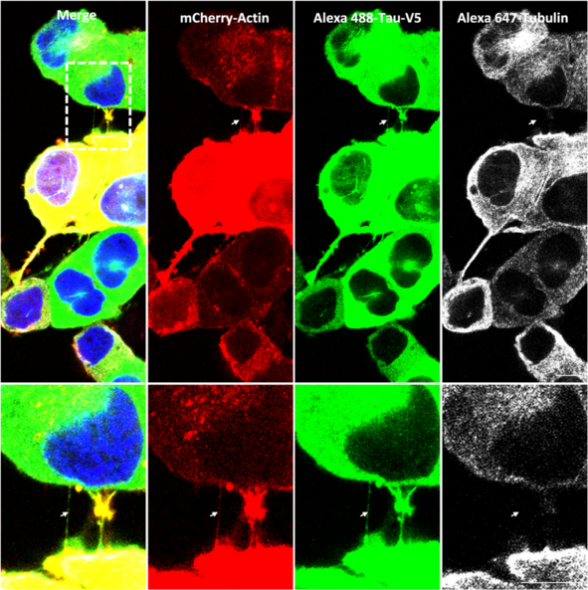
Tau is associated with TNTs in neuronal CAD cells. Cells were plated in Lab-Tek chamber slides and co-infected with LVs encoding mCherry-Actin (*red*) and V5-hTau1N4R (*green*). Neuronal CAD cells were processed for immunocytochemistry analysis using anti-V5 antibodies visualized with an Alexa 488-labeled secondary antibody (*green*) and anti-acetylated-tubulin visualized with an Alexa 647-labeled secondary antibody (*white*). Nuclei were labeled with DAPI (*blue*). Cells were imaged with an inverted laser-scanning confocal microscope using a 40× oil-immersion lens (NA 1.3), and the images were processed using ZEN and ImageJ. A focal plane was collected for each specimen. Scale bars: 10 μm. TNTs (*white arrows*), which are not always bound to the dish, are shown in enlargements

We next investigated the presence of Tau in TNTs in basal conditions in neuronal CAD cells. Tau containing a V5 epitope [19] was overexpressed using a lentiviral vector, and its presence in TNTs was tracked. V5 immunoreactivity was detected in TNTs established between neuronal CAD cells (Fig. 1), demonstrating the presence of soluble Tau within TNTs and co-localized with actin (Additional file 5: Figure S5 a and c; *n* = 3, Pearson correlation = 0.7 ± 0.09).

### Extracellular Tau species favors TNTs formation

**Fig. 2:**
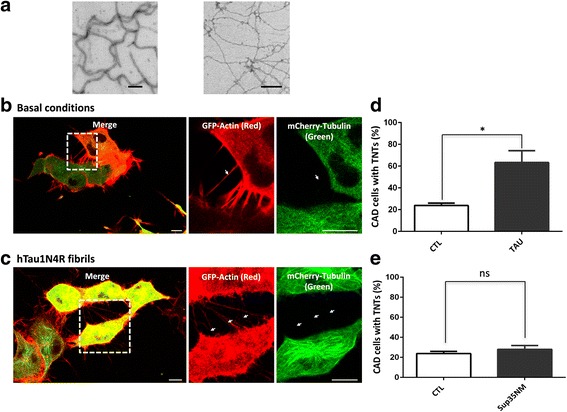
Extracellular Tau species activate TNT formation in neuronal CAD cells. **a** Characterization of Tau fibrils. Electron micrographs of recombinant hTau1N4R (40 μM) fibrils assembled in the presence of heparin (10 μM), left panel, Sup35NM (20 μM) fibrils, right panel). Scale bars: 0.1 μm. **b** Confocal imaging of TNTs in neuronal CAD cells in basal conditions. Cells were infected with LVs encoding GFP-Actin (r*ed*) and mCherry-Tubulin (*green*), and confocal imaging was performed 24 h later. Inset: high magnification of TNTs (*white arrows*). **c** Confocal imaging of TNTs in neuronal CAD cells after activation with Tau. Cells were infected with LVs encoding GFP-Actin (red) and mCherry-Tubulin (*green*). At 24 h later, non-labeled hTau1N4R fibrils were added to the extracellular medium, and cells were immediately imaged. Inset: high magnification of TNTs (white arrows). **d** Quantification of CAD cells with TNTs under basal conditions (CTL) and after activation with 1 μM hTau1N4R fibrils (Tau); *n* = 4 independent experiments; 200 cells per experiment (*, *p* < 0.1; Mann-Whitney test). **e** Quantification of CAD cells with TNTs under basal conditions (CTL) and after activation with the amyloid fibril prion Sup35NM (Sup35); *n* = 3 independent experiments; 200 cells per experiment (NS, non significant; Mann-Whitney test). For (**b**), (**c**), (**d**) and (**e**), cells were observed via laser-scanning confocal microscopy using a 40× oil-immersion lens (NA 1.3) and processed with ZEN and ImageJ software. A focal plane was collected for each specimen. Scale bars: 10 μm

**Fig. 3:**
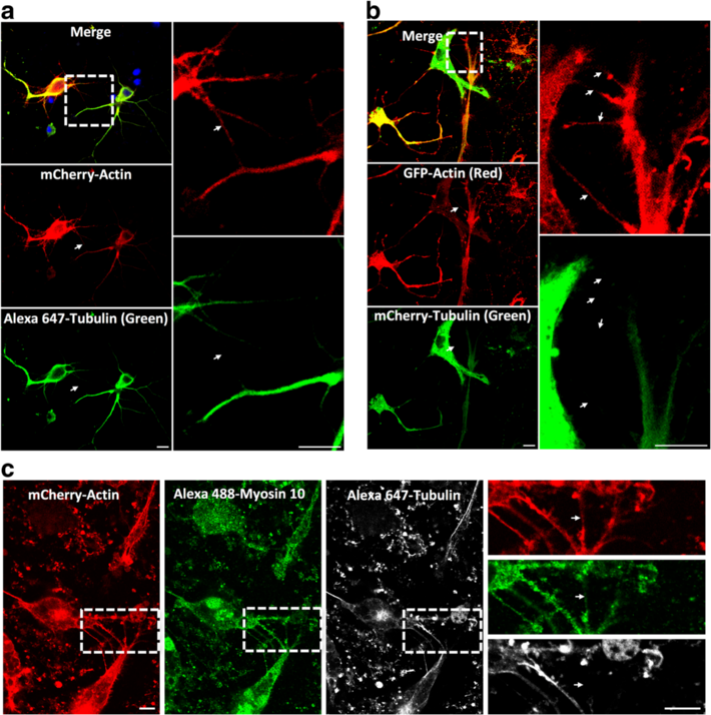
Extracellular hTau1N4R species favor the establishment of TNTs between primary neurons. **a** Imaging of TNTs in primary neurons. Cells were infected with LVs encoding mCherry-Actin (*red*). At 72 h post-infection, cells were processed for immunostaining analysis using anti-acetylated tubulin antibodies visualized with an Alexa 647-labeled secondary antibody (*green*, polymerized tubulin). Nuclei were labeled with DAPI (*blue*). **b** Real-time snapshots of TNTs in primary neurons co-infected with LVs encoding GFP-Actin (*red*) and mCherry-Tubulin (*green*, monomeric and polymerized tubulin). (**c**) Myo10 is present in TNTs in primary neurons. Neurons were plated in Lab-Tek chambers, infected with LVs encoding mCherry-Actin (*red*) and processed for immunocytochemistry analysis using anti-myosin 10 antibodies visualized with an Alexa 488-labeled secondary antibody (*green*) and anti-acetylated tubulin visualized with an Alexa 647-labeled secondary antibody (*white*). Images for (**a**), (**b**) and (**c**) were acquired via laser-scanning confocal microscopy using a 40× oil-immersion lens (NA 1.3) and processed with ZEN and ImageJ software. A focal plane was collected for each specimen. TNTs are shown in enlargements. For (**a**), (**b**) and (**c**), to observe TNTs in primary neurons, 1 μM hTau1N4R fibrils were added in extracellular medium. TNTs were never observed in primary neurons without addition of exogenous Tau fibrils. Scale bars: 10 μm

TNTs formation is still ill-defined [28–34] however stresses including protein fibrils [8, 12, 35], virus infection [6, 7, 31] and oxidation [12] have been reported to trigger their formation. We therefore evaluated whether extracellular Tau species (monomers and fibrils) might be a signal to trigger TNT formation. Monomeric full-length Tau was assembled into fibrils (Fig. 2a) as described in the methods section. The resulting assemblies were diluted in the growth medium of neuronal CAD cells, and TNTs were quantified. Whereas the number of TNTs per cell is not modified, the the proportion of cells establishing TNTs reaches 63 % in the presence of extracellular Tau species versus 24 % in basal conditions (Fig. 2b-d, 2.6-fold increase, *p* < 0.05). This increase is specific to extracellular Tau species, as no such increase in TNTs number was observed in the presence of unrelated amyloid fibrils, such as the protein Sup35NM from Saccharomyces cerevisiae [22] that binds to mammalian cells [36, 37] where it displays prion properties [38] (Fig. 2e, *p* = 0.45). We then investigated the ability of extracellular Tau species to induce TNT formation in primary neurons. Similarly to our observations in neuronal CAD cells (Fig. 2), extracellular Tau species in the culture medium activated TNT formation as assessed by their specific tubulin-negative (monomeric or polymerized) and actin- and myosin-10-positive staining (Fig. 3).

### The protein Tau is a constitutive component of TNTs

**Fig. 4:**
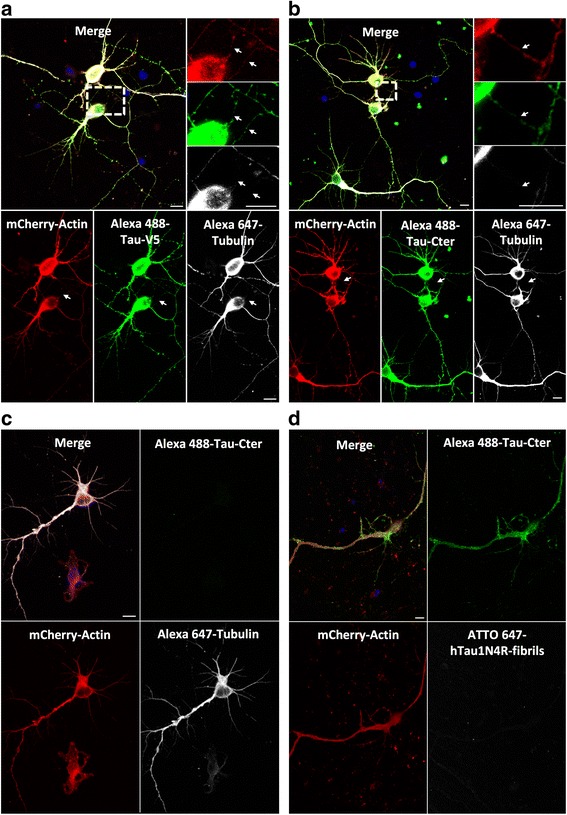
Tau is found in TNTs in primary neurons. **a** Neurons were infected with LVs encoding mCherry-Actin and V5-hTau1N4R. At 72 h post-infection, cells were processed for immunostaining analysis using anti-V5 antibodies visualized with an Alexa 488-labeled secondary antibody (*green*) and anti-acetylated tubulin visualized with an Alexa 647-labeled secondary antibody (*white*). **b** Acquisition of TNTs connecting primary neurons and containing endogenous Tau protein. Cells were infected with LVs encoding mCherry-Actin. At 72 h post-infection, neurons were processed for immunostaining analysis using anti-C-Terminal Tau antibodies (Tau-Cter) visualized with an Alexa 488-labeled secondary antibody (*green*) and anti-acetylated tubulin visualized with an Alexa 647-labeled secondary antibody (*white*). For (**a**) and (**b**), TNTs containing Tau are shown in enlargements (*white* arrows). **c** Cells were infected with LVs encoding mCherry-Actin. At 72 h post-infection, neurons were processed for immunocytochemistry using anti-acetylated tubulin visualized with an Alexa 647-labeled secondary antibody (*white*) and anti C-Terminal Tau antibodies (Tau-Cter) visualized with an Alexa 488-labeled secondary antibody (*green*). The C-Terminal Tau antibodies used were saturated with Tau proteins for 24 h at 4 °C to block the specific fluorescence signal of tau. Nuclei were labeled with DAPI (*blue*). For (**a**), (**b**) and (**c**), 1 μM hTau1N4R fibrils were added in the extracellular medium. (d) Cells were infected with LVs encoding mCherry-Actin. TNT formation was activated by ATTO 647-hTau1N4R fibrils. At 72 h later, neurons were processed for immunocytochemistry using anti C-Terminal Tau antibody (Tau-Cter) visualized with an Alexa 488-labeled secondary antibody (*green*). For (**a**), (**b**), (**c**) and (**d**), images were acquired using laser-scanning confocal microscopy with a 40× oil-immersion lens (NA 1.3) and processed with ZEN and ImageJ software. Images in (**b**) and (**c**) were acquired with the same optical settings and a focal plane was collected. Nuclei were labeled with DAPI (b*lue*). Scale bars: 10 μm

We next designed experiments to determine whether soluble Tau is present within TNTs. Tau containing a V5 epitope was overexpressed as described above, and its presence in TNTs was assessed. V5 immunoreactivity was detected in TNTs formed between primary neurons (Fig. 4a), demonstrating the presence of soluble Tau in these cellular extensions. Because the presence of Tau in TNTs might be due to the over-expression system used, we also labeled the murine endogenous Tau in primary neurons rather than in CAD cells, which express endogenous Tau at an undetectable level (Additional file 1: Figure S1 a-c). Immunostaining using antibodies directed against a Tau C-terminal moiety (Tau C-ter) and acetylated tubulin showed Tau C-ter immunoreactivity in TNTs (Fig. 4b). Saturation of anti-Tau C-ter antibodies with monomeric Tau switched off the fluorescence signal (Fig. 4c), demonstrating the specificity of endogenous Tau staining with the anti-Tau C-ter antibodies. To demonstrate that the signal we detected is not due to residual exogenous fibrillar Tau that had been used to trigger TNT formation, we repeated the staining after exposing primary neurons to Tau-ATTO 647 fibrils. No yellow fluorescence in TNTs, i.e., no colocalization of endogenous (green) immunoreactivity and exogenous Tau (far red fluorescence), was observed (Fig. 4d), indicating that soluble Tau is a constitutive component of TNTs. As shown in neuronal CAD cells (Additional file 5: Figure S5 a and c), Tau also co-localized with actin in TNTs in primary neurons (*n* = 3, Pearson correlation = 0.7 ± 0.1) (Additional file 5: Figure S5 b and c).

### Trafficking of fibrillar Tau from cell to cell through TNTs

TNTs have been shown to be involved in the spread of various pathogens and protein fibrils implicated in neurodegenerative and infectious diseases [6, 9, 11, 13, 39, 40]. In AD, Tau pathology progresses through a hierarchical pathway, but the Tau species involved in cell-to-cell propagation remains unclear. We therefore assessed the presence of exogenous fibrillar Tau species within TNTs in CAD neuronal cells and primary neurons.

**Fig. 5:**
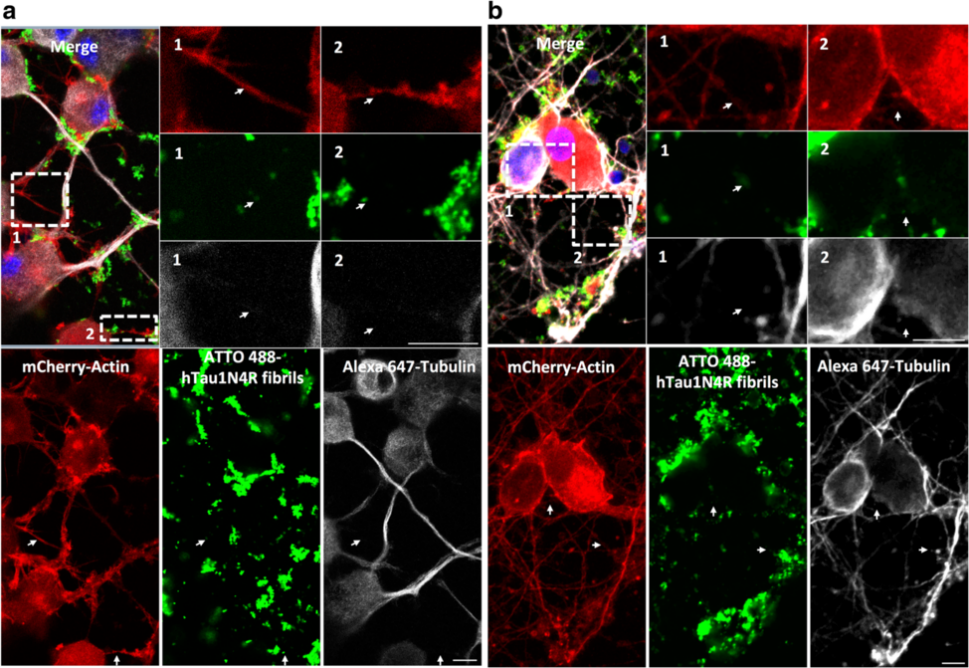
Exogenous Tau fibrils are found within TNTs. CAD neuronal cells (**a**) or primary neurons (**b**) were plated in Lab-Tek chamber slides and infected with LVs encoding mCherry-Actin (red). At 48 h post-infection, cells were incubated for six hours with extracellular ATTO 488-hTau1N4R fibrils/liposomes preparation (*green*). Cells were processed for immunostaining analysis using an anti-acetylated tubulin visualized with an Alexa 647-labeled secondary antibody (*white*). Nuclei were labeled with DAPI (*blue*). TNTs containing Tau are shown in enlargements (white arrows). For acquisition, a focal plane was collected for specimen. Images were acquired with an inverted laser-scanning confocal microscope using a 40× oil-immersion lens (NA 1.3) and processed using the programs ZEN and ImageJ. Scale bars: 10 μm

**Fig. 6:**
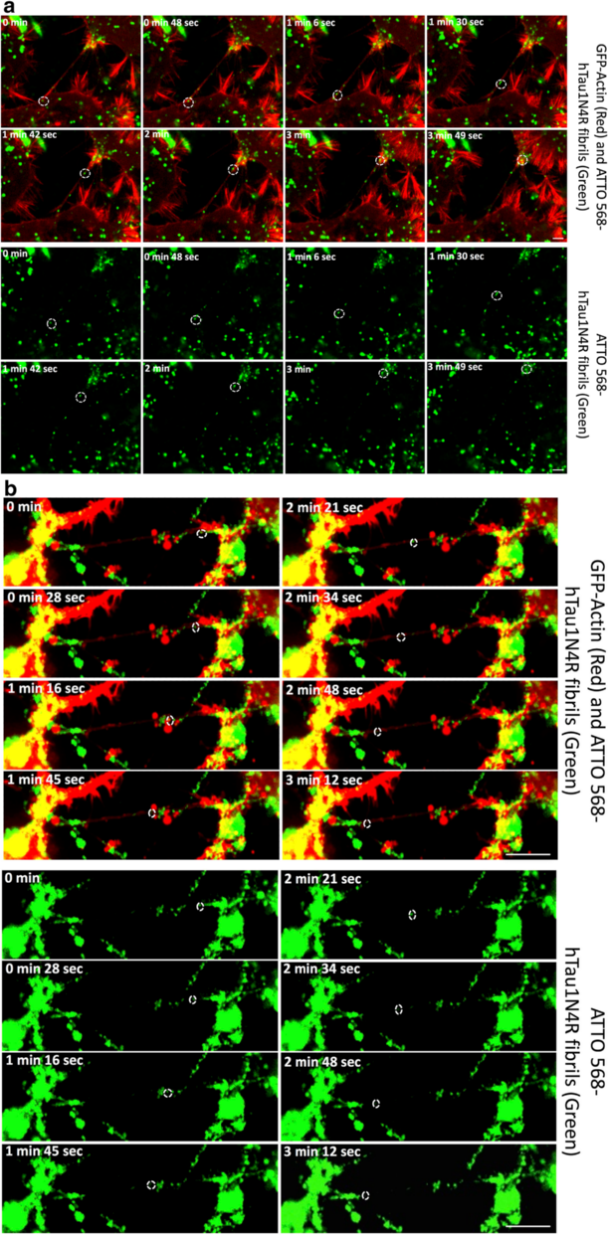
Neuron-to-neuron transfer of Tau fibrils through TNTs. For (**a**) and (**b**), cells were infected with LVs encoding GFP-Actin (*red*). At 48 h post-infection, cells were incubated for six hours with 1 μM ATTO 568-hTau1N4R fibrils (*green*). **a** Snapshots selected from a time-lapse video and placed in a gallery to visualize neuron-to-neuron transfer of extracellular Tau fibrils by TNTs in neuronal CAD cells. Time-lapse videos were acquired during 5 h and 33 min with an inter-image interval of 6 s. **b** Images selected from a video to visualize transfer of extracellular hTau1N4R fibrils by TNTs in primary neurons. Neurons were imaged every 6 s for 51 min. For (**a**) and (**b**), cells were filmed with an inverted spinning disk microscope using a 63× oil-immersion lens (NA 1.4) and processed with ZEN *blue* and ImageJ software. For time-lapse acquisition, a focal plane was collected for each specimen. Scale bars: 10 μm

**Fig. 7:**
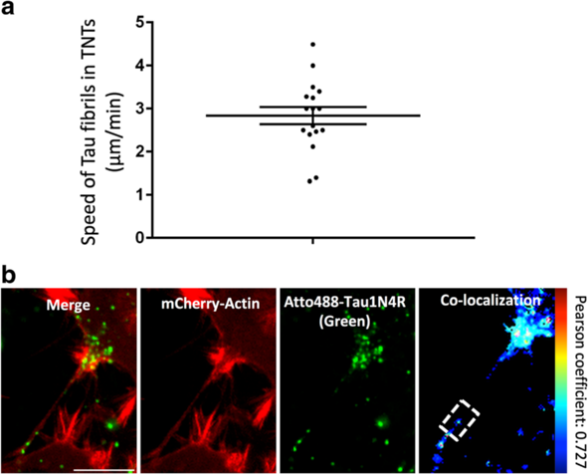
Characteristics of Tau fibrils trafficking in TNTs. For (**a**) and (**b**), cells were infected with LVs encoding GFP-Actin (*red*). At 48 h post-infection, cells were incubated for six hours with 1 μM ATTO 568-hTau1N4R fibrils (*green*). a The speed of Tau fibrils in TNTs in CAD neuronal cells was calculated from 17 independent videos. **b** Co-localization analysis between Tau fibrils and actin in TNTs. Significant positive correlation was found between Tau fibrils and actin (Pearson correlation coefficient: 0.727). For (**a**) and (**b**), cells were filmed with an inverted spinning disk microscope using a 63× oil-immersion lens (NA 1.4) and processed with ZEN blue and ImageJ software. A focal plane was collected for each specimen Scale bar: 10 μm


To visualize Tau fibrils inside TNTs, cells were treated 6 h with labeled exogenous hTau1N4R. Tau fibrils were observed within neuronal CAD cells (Fig. 5a and Additional file 6: Figure S6) and primary neurons (Fig. 5b) TNTs. Time-lapse analysis over a period of 10 h revealed Tau fibrils, moving inside TNTs in both neuronal CAD cells (Fig. 6a and Additional file 7: Figure S7 a, b and c) and primary neurons (Fig. 6b and Additional file 7: Figure S7 d, e and f). The mean speed of Tau in TNTs was calculated from video microscopy, showing that Tau was moving from donor to recipient cells inside these structures at a mean speed of 2.83 ± 1.99 μm/min (Fig. 7a, *n* = 17), displacements that are compatible with actin-based motility (typically 3 μm/min) [41]. In addition and as demonstrated for soluble Tau (Additional file 5: Figure S5), Tau fibrils co-localize with actin in TNTs (Fig. 7c, *n* = 3, Pearson correlation = 0.72 ± 0.1).

Three-dimensional view of exogenous Tau fibrils within TNTs. Neuronal CAD cells were plated in Lab-Tek chamber slides and infected with LVs encoding mCherry-Actin (red). 48 h postinfection, cells were incubated six hours with extracellular ATTO 488-hTau1N4R fibrils (green). Cells were processed for immunostaining analysis using an anti-acetylated tubulin visualized with an Alexa 647-labeled secondary antibody (white). Nuclei were labeled with DAPI (blue). TNTs containing Tau are shown by three-dimensional view. Images (focal series) were acquired with an inverted laser-scanning confocal microscope using a 40× oil-immersion lens (NA 1.3) and processed using the programs ZEN and ImageJ. (MOV 2405 kb)



Movie for neuron-to-neuron transfer of extracellular Tau fibrils by TNTs in neuronal CAD cells. (MOV 1078 kb)



Movie for neuron-to-neuron transfer of extracellular Tau fibrils by TNTs in neuronal CAD cells. (MOV 894 kb)



Movie of in vitro tracking of extracellular Tau fibrils in TNTs in CAD cells. (MOV 2307 kb)



Movie for neuron-to-neuron transfer of extracellular hTau1N4R fibrils by TNTs in primary neurons. (MOV 148 kb)



Movie for neuron-to-neuron transfer of extracellular hTau1N4R fibrils by TNTs in primary neurons. (MOV 239 kb)



Movie of in vitro tracking of extracellular Tau fibrils in TNTs in primary neurons. Tracking were performed with the mtrack plugin of ImageJ. (MOV 596 kb)


## Discussion

Tau protein, a microtubule-associated protein first described in 1975, has long been regarded as a mono-functional intracellular protein that modulates neuronal microtubule dynamics via complex regulation of its phosphorylation state [42–44]. The role of this protein is widely documented in neurodegenerative diseases collectively known as tauopathies [45]. These diseases, including AD, are characterized by intracellular accumulation of fibrillar material and neuronal loss. Through a set of neuroanatomical and biochemical studies, a spatio-temporal progression of Tau aggregation has been identified, thereby defining pathological stages as reported for example in AD [46–48].

There is now a growing body of evidence that Tau is a multifunctional protein involved in various pathophysiological processes that were previously unsuspected. These new functions related to specific locations (nucleus [49–52], plasma membrane [53, 54], synapses [55, 56]…) of Tau protein may help in the design of innovative therapeutic approaches. In addition, Tau has been identified as physiologically secreted into the extracellular space [57, 58]. Moreover, the presence of Tau in cerebrospinal fluid under pathological conditions has been known for many years [59], and in stress conditions, it has recently been identified in extracellular compartments such as cell culture media [60, 61] or interstitial fluids [62]. These data, combined with the existence of a hierarchical progression of Tau pathology, recently led to the hypothesis that Tau assemblies have prion-like properties. Tau assemblies propagate from affected neuronal cells to naïve neuronal cells [63–65]. To traffic between cells, they must either be secreted naked or be encapsulated into vesicles. Naked Tau assemblies can be secreted into the extracellular environment by exocytosis and taken up by naïve cells by endocytosis. Evidence for the secretion of Tau species within extracellular vesicles e.g., exosomes (~30–100 nm), which originate from the fusion of multivesicular bodies with the plasma membrane, and ectosomes (~100–1000 nm), which form directly from the plasma membrane, has been reported, but the contribution of these systems to the spread of disease remains to be confirmed and defined [65–68].

Previous studies have reported the presence of misfolded protein assemblies associated with disease within TNTs directly connecting the cytoplasm of distant cells, from immune to neuronal cells [1]. Indeed, in addition to allowing the long-range intercellular transfer of cytoplasmic macromolecules, plasma membrane components, vesicles and organelles, TNTs have been shown to allow the transfer of pathogens such as HIV, of infectious prion particles, and of huntingtin-Exon1 aggregates from affected donor to naive recipient cells [7, 8, 13].

Here, we demonstrate that extracellular Tau species (monomers and fibrils) activate the formation of TNTs (Figs. 2 and 3) that subsequently facilitate fibrillar Tau transfer from neuron to neuron (Figs. 4, 5 and 6). Extracellular Tau is found in stress and pathological conditions [59–62] and may likely act as a signal for TNT formation. More interestingly, TNTs facilitate the transfer of Tau assemblies between neurons. While these assemblies are fibrillar in our setup, the Tau species involved in inter-neuronal propagation in vivo remain unclear and subject to debate [69]. Both oligomeric and fibrillar forms are found during the course of Tau pathology. Thus, one can speculate that both forms are involved in the process, albeit with different kinetics and functions. Nonetheless, our data clearly demonstrate that fibrillar Tau transfer from neuron to neuron through TNTs.

Our finding that Tau is present in TNTs is also of great importance. Indeed, TNTs are ill-defined, and various types of TNT-like structures have been described [26]. Identification and characterization of the types of tubular bridges that connect different cells (e.g., TNTs, filopodia) requires for specific markers. Our data demonstrate that Tau, together with actin, is a specific constitutive marker of TNTs. Our data further suggest that Tau may contribute to TNT formation and function, thus allowing a better characterization and understanding of these highly dynamic structures. This is in agreement with recent data showing that Tau organizes the actin networks. Indeed, in the absence of Tau, only single actin filaments could be observed, whereas in the presence of Tau, they progressively formed long and thick F-actin bundles [70, 71]. This actin-binding property of Tau, in combination with data emerging from this study, strongly suggests that Tau might be transported into TNTs via actin. However, Tau overexpression itself is not sufficient to induce TNT formation. Whether myosin 10, also found in neuronal TNTs and acting as an actin motor protein, is required remains to be elucidated. Identifying cellular mechanisms supporting in vivo pathological Tau transfer through TNTs will help us to define therapeutic targets. In fact, the role of TNTs in a pathological context such as inflammation has already been shown between widely spaced dendritic cells in the adult mouse cornea [28, 72]). Nevertheless, even if in different in vitro and in vivo experimental models [6, 7, 13, 14, 72], there is now a strong evidence for the role of TNTs in different stress-induced pathological processes including the transport of many pathological proteins and pathogens, it has never been addressed in vivo in the brain and remains to be investigated.

## Conclusions

To conclude, by enhancing TNT formation, exogenous Tau species appears to mediate a deleterious cycle that favors the transfer of assemblies tightly associated with disease between neurons.

## Additional files

Additional file 1: Figure S1. Endogenous Tau in neuronal CAD cells and fluorescence setup controls. (a) Neuronal CAD cells were infected with LV encoding mCherry-Actin (red). Cells were processed for immunostaining analysis using anti-C-Terminal Tau antibodies (Tau-Cter) visualized with an Alexa 488-labeled secondary antibody (green) and anti-acetylated tubulin visualized with an Alexa 647-labeled secondary antibody (white). (b) Cells were infected with LV encoding mCherry-Actin and processed for immunostaining analysis using anti-C-Terminal Tau antibodies (Tau-Cter) visualized with an Alexa 488-labeled secondary antibody (green) and anti-acetylated tubulin visualized with an Alexa 647-labeled secondary antibody (white). The Tau antibody used were saturated with Tau proteins for 24 h at 4 °C to block the specific fluorescence signal of tau. Nuclei were labeled with DAPI (blue). (c) Biochemical analysis of endogenous Tau in neuronal CAD cells. Cell lysates were analyzed by immunoblotting using Tau-Cter and Tau-Nter antibodies. Controls correspond to mouse hippocampus cell homogenate (CTL1) and Tau protein overexpressed in cells using LVs encoding Tau (CTL 2). (d) Setup for visualization of the non-specific fluorescence signal of secondary antibodies or fusion proteins (GFP or mCherry) in neuronal CAD cells. (e) Setup for visualization of non-specific fluorescence signal of secondary antibodies or fusion protein (GFP or mCherry) in primary neurons. For (d) and (e), cells were incubated with secondary antibodies (Alexa 488 or Alexa 647). Nuclei were labeled with DAPI (blue). For images, a focal plane was collected for specimen. Scale bars: 10 μm. (TIFF 12495 kb)
Additional file 2: Figure S2. Characterisation of Tau 1N4R fibrillar assemblies. (a) Representative negatively stained TEM of sonicated fibrils. Scale bar, 100 nm. (b) Length distribution of sonicated Tau fibrils obtained by measuring the length of 227 fibrils in negatively stained TEM samples. (TIF 19797 kb)
Additional file 3: Figure S3. Phenotypical characterization of TNTs established between neuronal CAD cells. (a) Maximum-intensity projection of TNTs in CAD neuronal cells. CAD cells were plated in Lab-Tek chamber slides, infected with LVs encoding mCherry-Actin (red) and processed for immunostaining analysis using anti-acetylated tubulin antibodies visualized with an Alexa 488-labeled secondary antibody (green, polymerized tubulin). Nuclei were labeled with DAPI (blue). (b) Real-time focal plane acquisition of TNTs in CAD cells infected with LV encoding mCherry-Actin (red) and incubated with tubulin tracker (Taxol, green, polymerized tubulin). (c) Real-time focal plane acquisition of TNTs in CAD cells co-infected with LVs encoding GFP-actin (red) and mCherry-Tubulin (green, monomeric and polymerized tubulin). (d) Myo10 is present in TNTS in CAD neuronal cells. CAD cells were plated in Lab-Tek chamber slides, infected with LV encoding mCherry-Actin (red) and processed for immunocytochemistry analysis using anti-myosin 10 antibodies visualized with an Alexa 488-labeled secondary antibody (green). Tubulin is visualized using an anti-acetylated tubulin antibody and an Alexa 647-labeled secondary antibody (white). For acquisition, a focal plane was collected for specimen. Images in (a), (b), (c) and (d) were acquired using an inverted laser-scanning confocal microscope using a 40× oil-immersion lens (NA 1.3) and processed with ZEN and ImageJ software. TNTs (white arrows), which are not always bound to the dish, are shown in enlargements. Experiments were replicated at least three times. Scale bars: 10 μm. (TIFF 21472 kb)
Additional file 4: Figure S4. Size and formation of TNTs in neuronal CAD cells. (a) Length and diameter of TNTs in neuronal CAD cells in fixed and live cells. Twenty TNTs (white arrow) were analyzed using mCherry-Actin (red) and anti-acetylated tubulin antibodies visualized with an Alexa 488-labeled secondary antibody (green) (fixed cells) or GFP Actin and mCherry-Tubulin (live cells). (***, *p* < 0.001; Mann-Whitney test). (b) Neuronal CAD cells were infected with LVs encoding GFP-Actin (Red) and mCherry-Tubulin (Green) and filmed with an inverted Nikon microscope using a 40× air-immersion lens (NA 0.9) and processed with NIS software. Still images were selected from time-lapse videos and placed in a gallery to visualize the TNT “filopodia-like” formation mechanism. Cell 1 extends a tube in the direction of cell 2. The tube of cell 1 docks to cells 2 and creates a TNT bridge (white arrows). Time-lapse images for this series were acquired during 1 h with an inter-image interval of 17.28 s. (c) Neuronal CAD cells were infected with LVs encoding mCherry-Actin and incubated with tubulin tracker (taxol, green). Real time focal plane observation of cells was performed by laser-scanning confocal microscopy using a 40× oil-immersion lens (NA 1.3) and processed with ZEN and ImageJ software. Snapshots from a video were selected to show the “kiss-and-run” formation mechanism of TNTs. Cells 1 and 2 are coming closer and moving forward to develop TNT bridges (white arrows). Cells were imaged every 25 s for 13 min. Scale bars: 10 μm. (d) Movie for “filopodia-like” formation mechanism in neuronal CAD cells. Cells were co-infected with LVs encoding GFP-Actin and mCherry-Tubulin. After 24 h, cells were observed with an inverted Nikon microscope using a 40× air-immersion lens (NA 0.9) and processed with NIS and ImageJ software. Cells were imaged every 17.28 s for 1 h. Only GFP-actin (white) is presented. (e) Movie for “kiss-and-run” formation mechanism in neuronal CAD cells. Cells were infected with LVs encoding mCherry-Actin and incubated with a tubulin tracker (taxol, green). Real time observations were performed using a laser-scanning confocal microscope and a 40× oil-immersion lens (NA 1.3) and were processed with ZEN and ImageJ software. Cells were imaged every 25 s for 13 min. Only mCherry-actin (white) is presented. (TIFF 16089 kb)
Additional file 5: Figure S5. Tau and actin co-localize in TNTs. (a) and (b) Co-localization analysis between Tau and actin in TNTs in CAD cells and primary neurons. (C) Pearson correlation coefficient analysis in CAD cells and primary neurons. Significant positive correlations were found between Tau and actin in CAD cells (*n* = 3, Pearson correlation coefficient = 0.764 ± 0.09) and primary neurons (*n* = 3, Pearson correlation coefficient = 0.772 ± 0.1). For (a) and (b), cells were plated in Lab-Tek chamber slides and co-infected with LVs encoding mCherry-Actin (red) and V5-hTau1N4R (green). Cells were processed for immunocytochemistry analysis using anti-V5 antibodies visualized with an Alexa 488-labeled secondary antibody (green) and anti-acetylated-tubulin visualized with an Alexa 647-labeled secondary antibody (white). Cells were imaged with an inverted laser-scanning confocal microscope using a 40× oil-immersion lens (NA 1.3), and the images were processed with ZEN and ImageJ software. Scale bars: 10 μm. (TIFF 7665 kb)
Additional file 7: Figure S7. Fibrillar Tau transfer through TNTs in neurons. (a) Movies for neuron-to-neuron transfer of extracellular Tau fibrils by TNTs in neuronal CAD cells. (b) Movie of in vitro tracking of extracellular Tau fibrils in TNTs in CAD cells. Tracking was performed with the mtrack plugin for ImageJ. (c) Kymograph representation of extracellular Tau fibrils inside TNTs in neuronal CAD cells. The kymograph was generated from the movie (b), which shows the fluorescence intensity along the x-axis over time (y-axis). (d) Movies for neuron-to-neuron transfer of extracellular hTau1N4R fibrils by TNTs in primary neurons. (e) Movie of in vitro tracking of extracellular Tau fibrils in TNTs in primary neurons. Tracking were performed with the mtrack plugin of ImageJ. (f) Kymograph representation of extracellular Tau fibrils inside TNTs in primary neurons. The kymograph was generated from the movie in (d), which shows the presence of fluorescence intensity along the x-axis over time (y-axis). For (c) and (f), the first acquisition of the time-lapse movie is the top of the kymograph (arrowhead 1), and the last acquisition is the bottom of the kymograph (arrowhead 2). Arrowheads represent the same object moving from the bottom to the top. For (a) and (d), cells were infected with LVs encoding GFP-Actin (red). At 48 h post-infection, cells were incubated six hours with ATTO 568-hTau1N4R fibrils (green). Cells were filmed with an inverted spinning disk microscope using 63× oil-immersion lens (NA 1.4) and processed using ZEN blue and ImageJ. For images, a focal plane was collected for specimen. Scale bars: 10 μm. (TIFF 16305 kb)
