# Supplementary figures and images for: Tunneling nanotube (TNT)-mediated neuron-to neuron transfer of pathological Tau protein assemblies
Source: Acta Neuropathol Commun. 2016 Nov 4;4:117. doi: 10.1186/s40478-016-0386-4 (PMC5096005; doi:10.1186/s40478-016-0386-4)

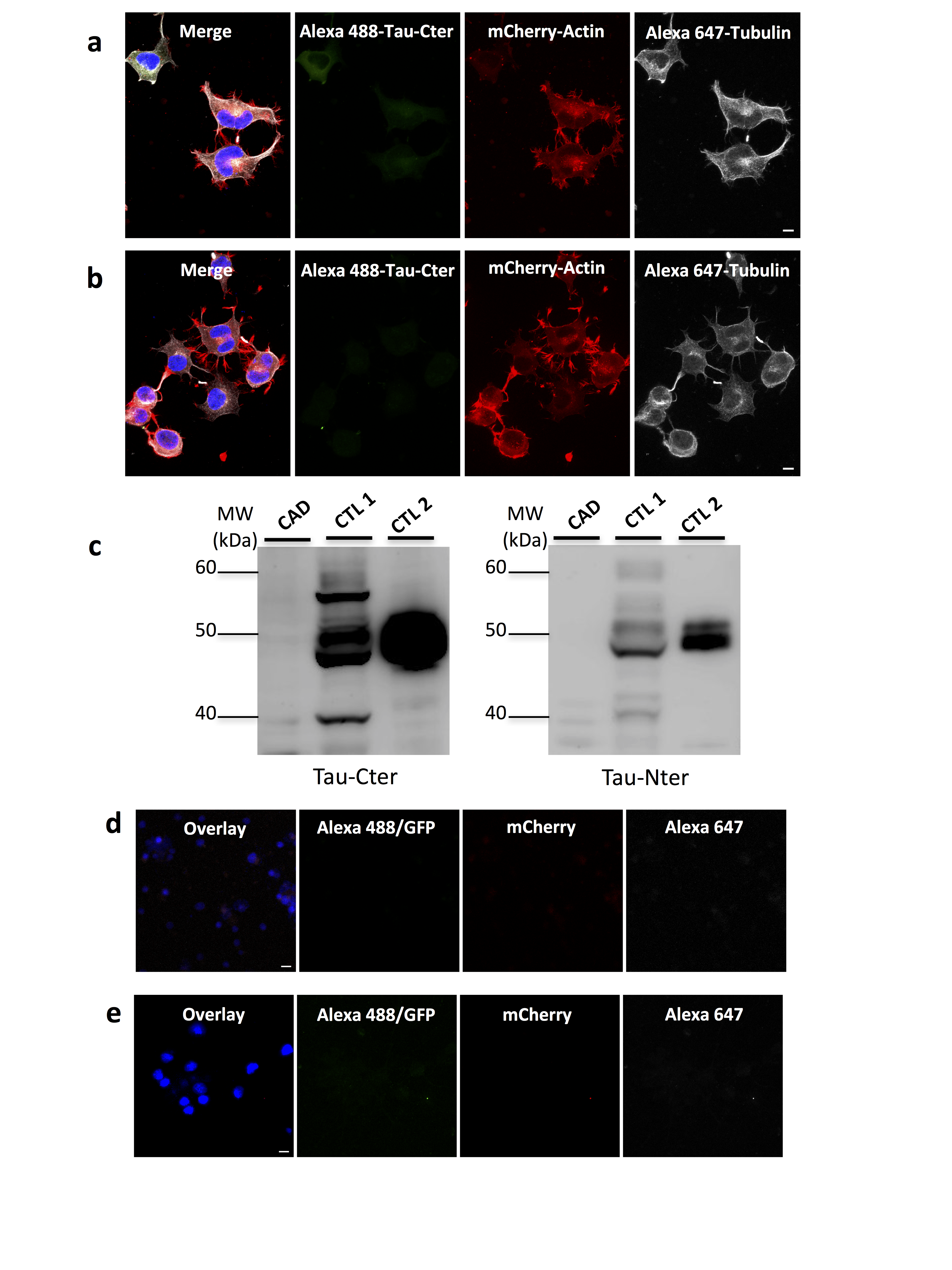

Supplement: Additional file 1: Figure S1. — Endogenous Tau in neuronal CAD cells and fluorescence setup controls. (a) Neuronal CAD cells were infected with LV encoding mCherry-Actin (red). Cells were processed for immunostaining analysis using anti-C-Terminal Tau antibodies (Tau-Cter) visualized with an Alexa 488-labeled secondary antibody (green) and anti-acetylated tubulin visualized with an Alexa 647-labeled secondary antibody (white). (b) Cells were infected with LV encoding mCherry-Actin and processed for immunostaining analysis using anti-C-Terminal Tau antibodies (Tau-Cter) visualized with an Alexa 488-labeled secondary antibody (green) and anti-acetylated tubulin visualized with an Alexa 647-labeled secondary antibody (white). The Tau antibody used were saturated with Tau proteins for 24 h at 4 °C to block the specific fluorescence signal of tau. Nuclei were labeled with DAPI (blue). (c) Biochemical analysis of endogenous Tau in neuronal CAD cells. Cell lysates were analyzed by immunoblotting using Tau-Cter and Tau-Nter antibodies. Controls correspond to mouse hippocampus cell homogenate (CTL1) and Tau protein overexpressed in cells using LVs encoding Tau (CTL 2). (d) Setup for visualization of the non-specific fluorescence signal of secondary antibodies or fusion proteins (GFP or mCherry) in neuronal CAD cells. (e) Setup for visualization of non-specific fluorescence signal of secondary antibodies or fusion protein (GFP or mCherry) in primary neurons. For (d) and (e), cells were incubated with secondary antibodies (Alexa 488 or Alexa 647). Nuclei were labeled with DAPI (blue). For images, a focal plane was collected for specimen. Scale bars: 10 μm. (TIFF 12495 kb) [file 40478_2016_386_MOESM1_ESM.tiff]

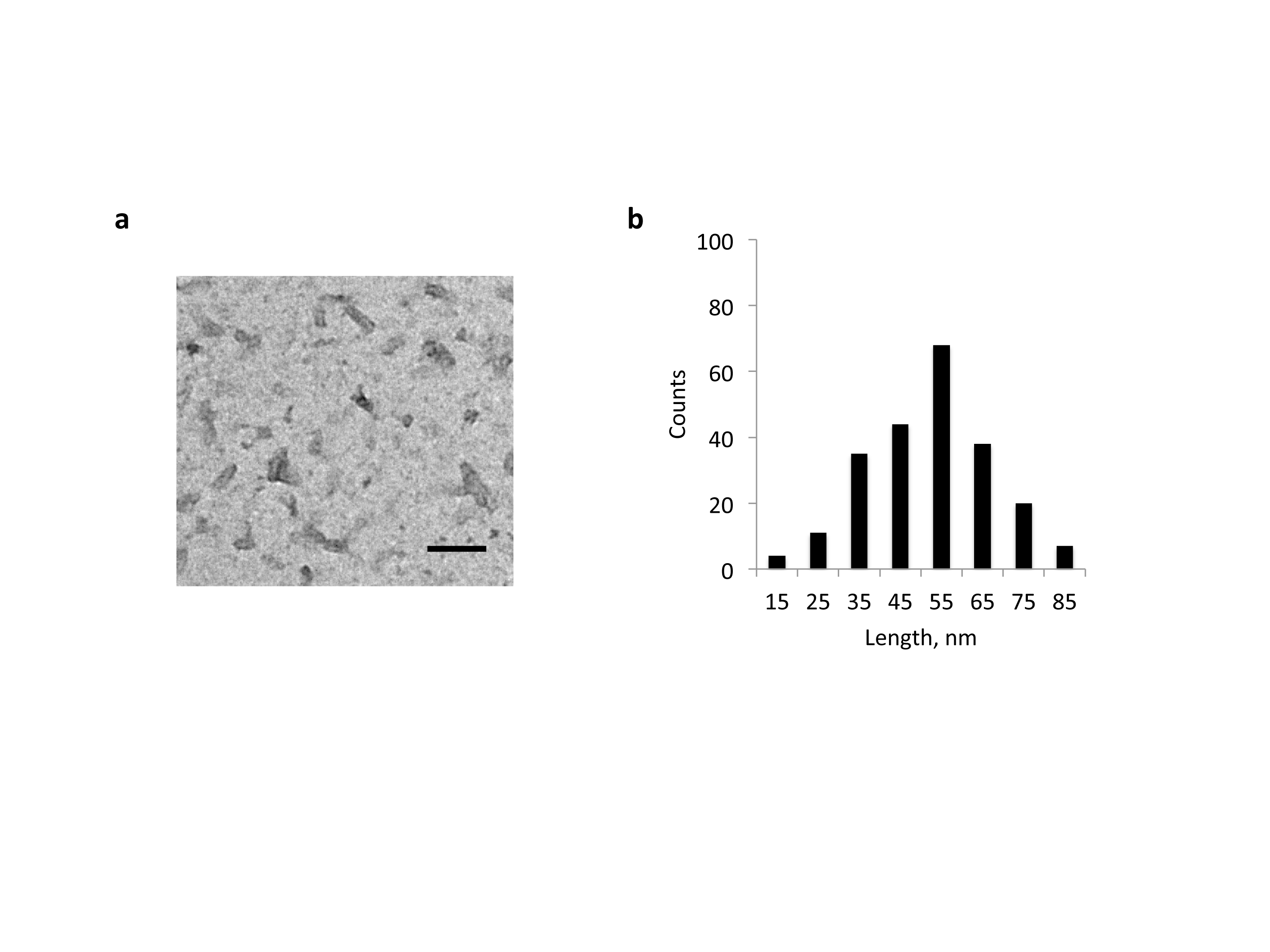

Supplement: Additional file 2: Figure S2. — Characterisation of Tau 1N4R fibrillar assemblies. (a) Representative negatively stained TEM of sonicated fibrils. Scale bar, 100 nm. (b) Length distribution of sonicated Tau fibrils obtained by measuring the length of 227 fibrils in negatively stained TEM samples. (TIF 19797 kb) [file 40478_2016_386_MOESM2_ESM.tif]

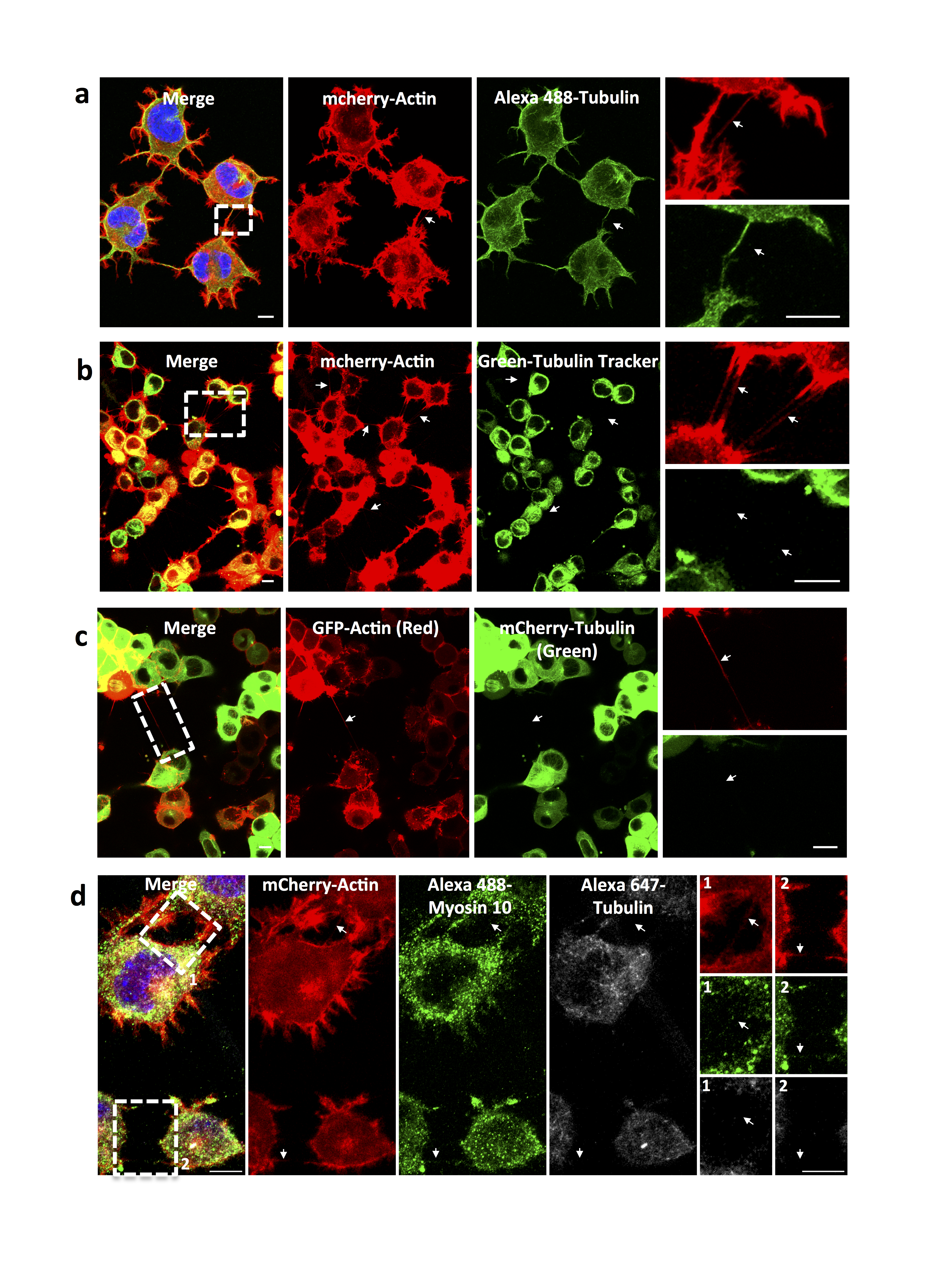

Supplement: Additional file 3: Figure S3. — Phenotypical characterization of TNTs established between neuronal CAD cells. (a) Maximum-intensity projection of TNTs in CAD neuronal cells. CAD cells were plated in Lab-Tek chamber slides, infected with LVs encoding mCherry-Actin (red) and processed for immunostaining analysis using anti-acetylated tubulin antibodies visualized with an Alexa 488-labeled secondary antibody (green, polymerized tubulin). Nuclei were labeled with DAPI (blue). (b) Real-time focal plane acquisition of TNTs in CAD cells infected with LV encoding mCherry-Actin (red) and incubated with tubulin tracker (Taxol, green, polymerized tubulin). (c) Real-time focal plane acquisition of TNTs in CAD cells co-infected with LVs encoding GFP-actin (red) and mCherry-Tubulin (green, monomeric and polymerized tubulin). (d) Myo10 is present in TNTS in CAD neuronal cells. CAD cells were plated in Lab-Tek chamber slides, infected with LV encoding mCherry-Actin (red) and processed for immunocytochemistry analysis using anti-myosin 10 antibodies visualized with an Alexa 488-labeled secondary antibody (green). Tubulin is visualized using an anti-acetylated tubulin antibody and an Alexa 647-labeled secondary antibody (white). For acquisition, a focal plane was collected for specimen. Images in (a), (b), (c) and (d) were acquired using an inverted laser-scanning confocal microscope using a 40× oil-immersion lens (NA 1.3) and processed with ZEN and ImageJ software. TNTs (white arrows), which are not always bound to the dish, are shown in enlargements. Experiments were replicated at least three times. Scale bars: 10 μm. (TIFF 21472 kb) [file 40478_2016_386_MOESM3_ESM.tiff]

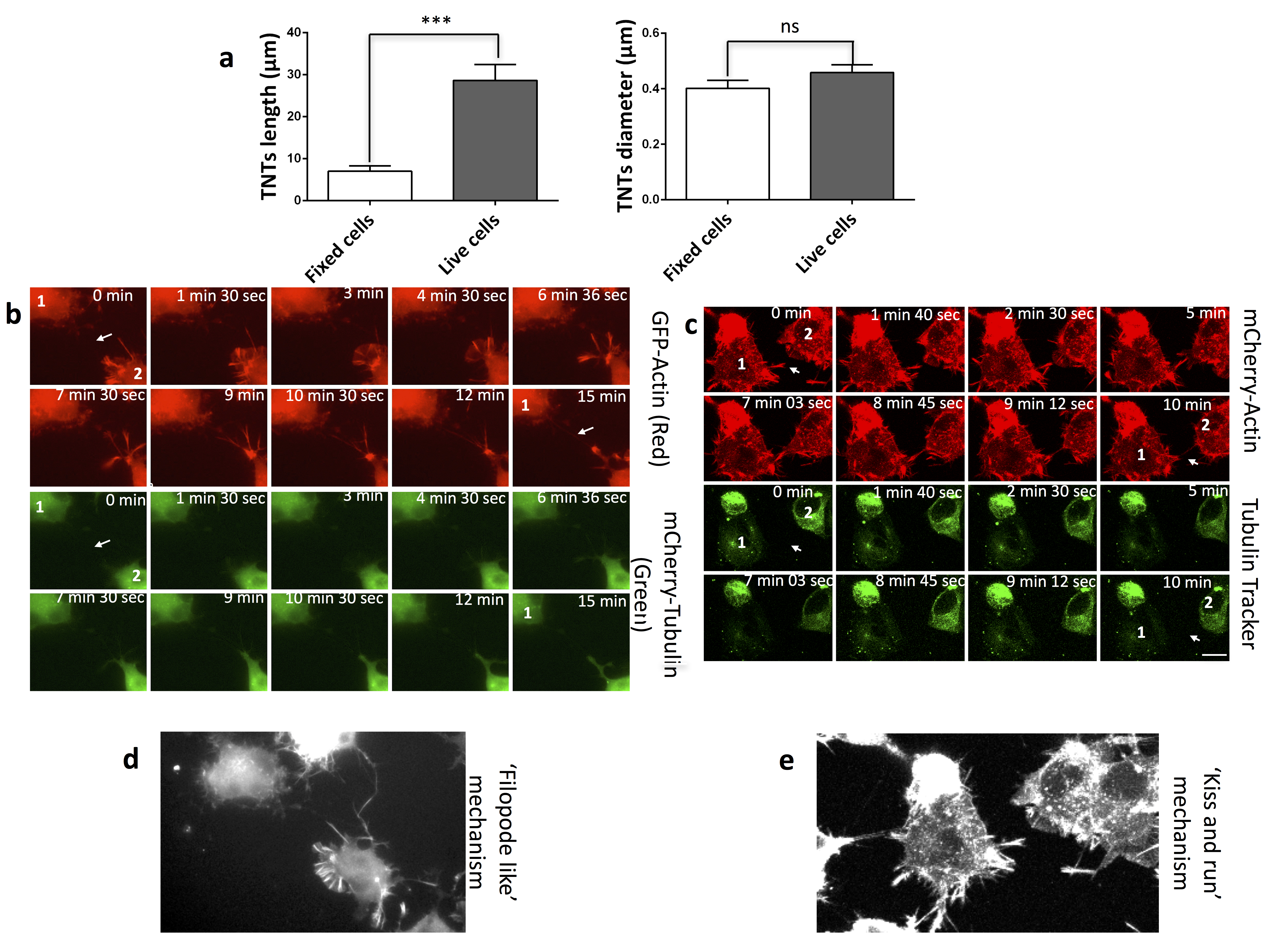

Supplement: Additional file 4: Figure S4. — Size and formation of TNTs in neuronal CAD cells. (a) Length and diameter of TNTs in neuronal CAD cells in fixed and live cells. Twenty TNTs (white arrow) were analyzed using mCherry-Actin (red) and anti-acetylated tubulin antibodies visualized with an Alexa 488-labeled secondary antibody (green) (fixed cells) or GFP Actin and mCherry-Tubulin (live cells). (***, p < 0.001; Mann-Whitney test). (b) Neuronal CAD cells were infected with LVs encoding GFP-Actin (Red) and mCherry-Tubulin (Green) and filmed with an inverted Nikon microscope using a 40× air-immersion lens (NA 0.9) and processed with NIS software. Still images were selected from time-lapse videos and placed in a gallery to visualize the TNT “filopodia-like” formation mechanism. Cell 1 extends a tube in the direction of cell 2. The tube of cell 1 docks to cells 2 and creates a TNT bridge (white arrows). Time-lapse images for this series were acquired during 1 h with an inter-image interval of 17.28 s. (c) Neuronal CAD cells were infected with LVs encoding mCherry-Actin and incubated with tubulin tracker (taxol, green). Real time focal plane observation of cells was performed by laser-scanning confocal microscopy using a 40× oil-immersion lens (NA 1.3) and processed with ZEN and ImageJ software. Snapshots from a video were selected to show the “kiss-and-run” formation mechanism of TNTs. Cells 1 and 2 are coming closer and moving forward to develop TNT bridges (white arrows). Cells were imaged every 25 s for 13 min. Scale bars: 10 μm. (d) Movie for “filopodia-like” formation mechanism in neuronal CAD cells. Cells were co-infected with LVs encoding GFP-Actin and mCherry-Tubulin. After 24 h, cells were observed with an inverted Nikon microscope using a 40× air-immersion lens (NA 0.9) and processed with NIS and ImageJ software. Cells were imaged every 17.28 s for 1 h. Only GFP-actin (white) is presented. (e) Movie for “kiss-and-run” formation mechanism in neuronal CAD cells. Cells were infected with LVs encoding m [file 40478_2016_386_MOESM4_ESM.tiff]

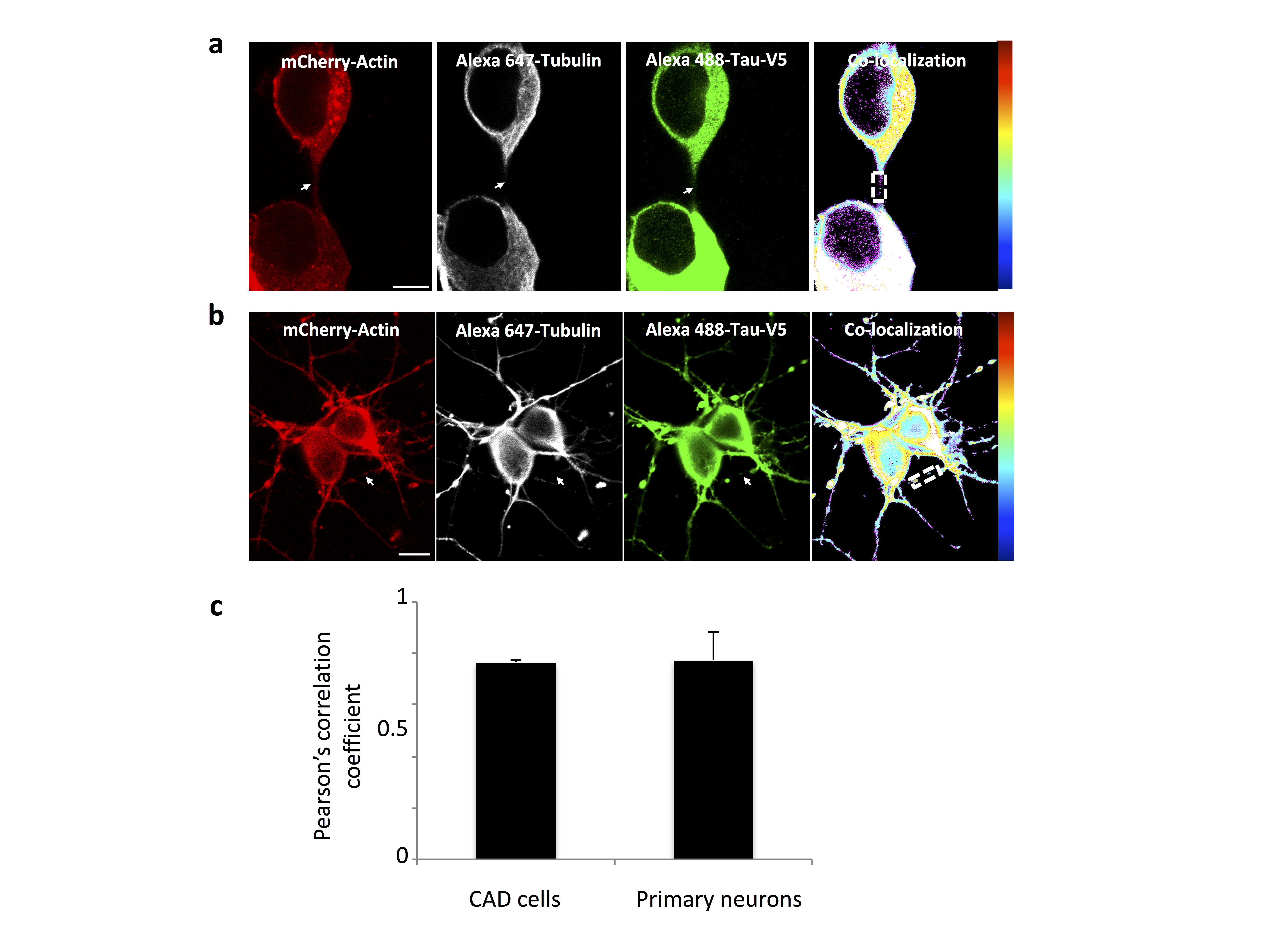

Supplement: Additional file 5: Figure S5. — Tau and actin co-localize in TNTs. (a) and (b) Co-localization analysis between Tau and actin in TNTs in CAD cells and primary neurons. (C) Pearson correlation coefficient analysis in CAD cells and primary neurons. Significant positive correlations were found between Tau and actin in CAD cells (n = 3, Pearson correlation coefficient = 0.764 ± 0.09) and primary neurons (n = 3, Pearson correlation coefficient = 0.772 ± 0.1). For (a) and (b), cells were plated in Lab-Tek chamber slides and co-infected with LVs encoding mCherry-Actin (red) and V5-hTau1N4R (green). Cells were processed for immunocytochemistry analysis using anti-V5 antibodies visualized with an Alexa 488-labeled secondary antibody (green) and anti-acetylated-tubulin visualized with an Alexa 647-labeled secondary antibody (white). Cells were imaged with an inverted laser-scanning confocal microscope using a 40× oil-immersion lens (NA 1.3), and the images were processed with ZEN and ImageJ software. Scale bars: 10 μm. (TIFF 7665 kb) [file 40478_2016_386_MOESM5_ESM.tiff]

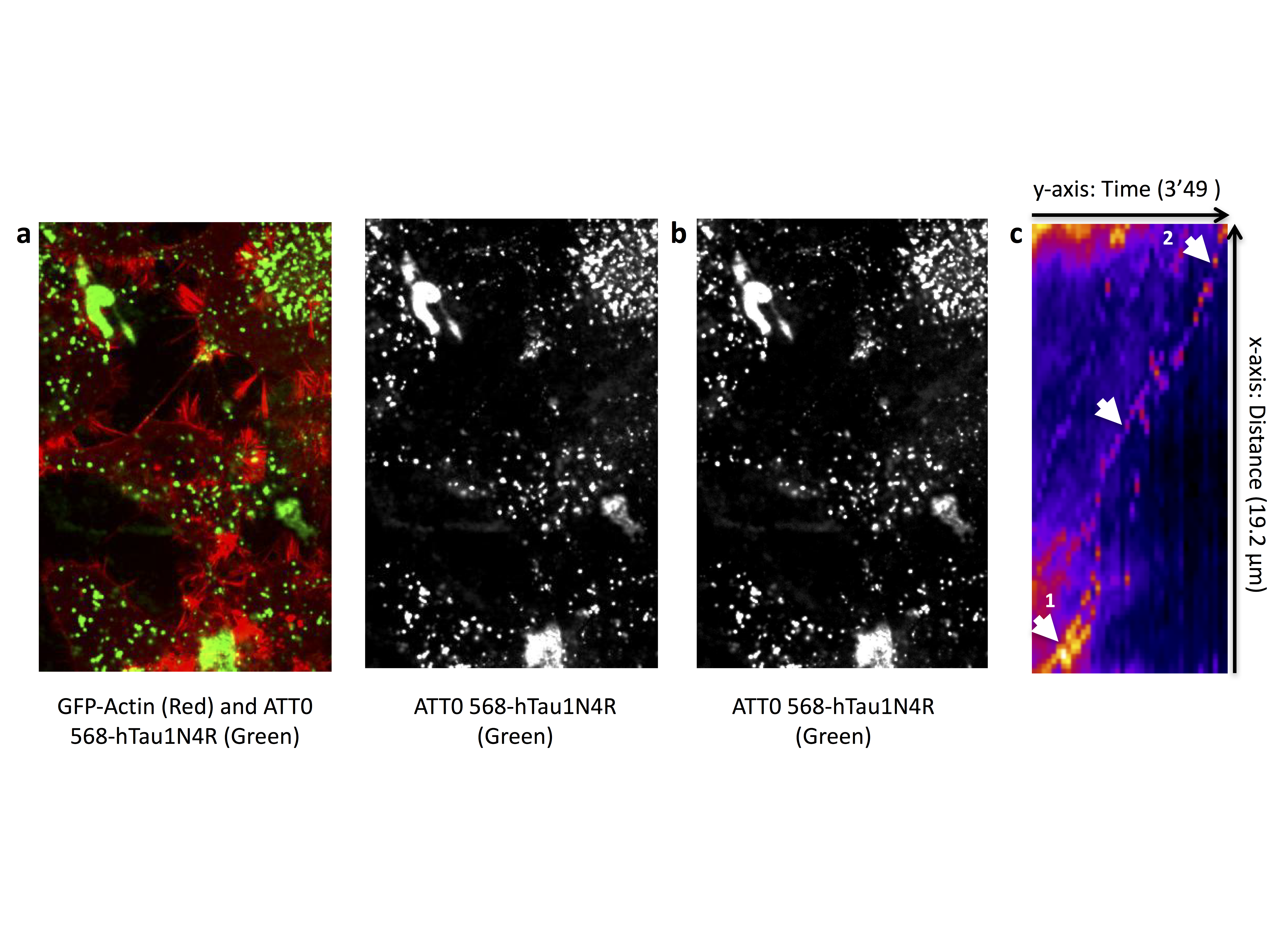

Supplement: Additional file 7: Figure S7. — Fibrillar Tau transfer through TNTs in neurons. (a) Movies for neuron-to-neuron transfer of extracellular Tau fibrils by TNTs in neuronal CAD cells. (b) Movie of in vitro tracking of extracellular Tau fibrils in TNTs in CAD cells. Tracking was performed with the mtrack plugin for ImageJ. (c) Kymograph representation of extracellular Tau fibrils inside TNTs in neuronal CAD cells. The kymograph was generated from the movie (b), which shows the fluorescence intensity along the x-axis over time (y-axis). (d) Movies for neuron-to-neuron transfer of extracellular hTau1N4R fibrils by TNTs in primary neurons. (e) Movie of in vitro tracking of extracellular Tau fibrils in TNTs in primary neurons. Tracking were performed with the mtrack plugin of ImageJ. (f) Kymograph representation of extracellular Tau fibrils inside TNTs in primary neurons. The kymograph was generated from the movie in (d), which shows the presence of fluorescence intensity along the x-axis over time (y-axis). For (c) and (f), the first acquisition of the time-lapse movie is the top of the kymograph (arrowhead 1), and the last acquisition is the bottom of the kymograph (arrowhead 2). Arrowheads represent the same object moving from the bottom to the top. For (a) and (d), cells were infected with LVs encoding GFP-Actin (red). At 48 h post-infection, cells were incubated six hours with ATTO 568-hTau1N4R fibrils (green). Cells were filmed with an inverted spinning disk microscope using 63× oil-immersion lens (NA 1.4) and processed using ZEN blue and ImageJ. For images, a focal plane was collected for specimen. Scale bars: 10 μm. (TIFF 16305 kb) [file 40478_2016_386_MOESM7_ESM.tiff]
